# Limited Secondary Transmission of the Novel Coronavirus (SARS-CoV-2) by Asymptomatic and Mild COVID-19 Patients in Bhutan

**DOI:** 10.4269/ajtmh.20-0672

**Published:** 2020-12-10

**Authors:** Tshokey Tshokey, Jamyang Choden, Kinley Dorjee, Pempa Pempa, Pema Yangzom, Wangdi Gyeltshen, Sonam Wangchuk, Tandin Dorji, Dechen Wangmo

**Affiliations:** 1 Jigme Dorji Wangchuck National Referral Hospital, Thimphu, Bhutan;; 2 Ministry of Health, Thimphu, Bhutan;; 3 Royal Center for Disease Control, Thimphu, Bhutan

## Abstract

As the COVID-19 pandemic continues, there is growing concordance and persisting conflicts on the virus and the disease process. We discuss limited transmissibility of the virus by asymptomatic and mild cases of COVID-19 patients in Bhutan. We followed up the secondary transmission of SARS-CoV-2 in the contacts of asymptomatic and mild COVID-19 patients in Bhutan. Bhutan had 33 confirmed COVID-19 cases in the country as of May 29, 2020. Of these, 22 (67%) were females. Except the first two cases (American tourists), the rest were Bhutanese living outside the country. The mean age of the Bhutanese patients was 26.3 (range 16–33) years. Close contacts of 27 of the 33 cases were followed up for signs and symptoms and COVID-19 positivity. The first two cases had 73 and 97 primary contacts, respectively, and equal number of secondary contacts (224). From the third case, a mandatory 21-day facility quarantine was instituted, all primary contacts were facility quarantined, and there were no secondary contacts. In total, the 27 cases had 1,095 primary contacts and 448 secondary contacts. Of these, 75 individuals were categorized as definite high-risk contacts. Secondary transmission occurred in seven high-risk contacts. Therefore, the overall secondary transmission was 9.0% (7/75) and 0.6% (7/1,095) among the high-risk and primary contacts, respectively. No transmission occurred in the secondary contacts. In contrast to several reports indicating high transmissibility of SARS-CoV-2 in contacts of confirmed cases, the mostly young, asymptomatic, and mild cases of COVID-19 in Bhutan showed limited secondary transmission.

## INTRODUCTION

The COVID-19 caused by the SARS-CoV-2 started as a cluster of unexplained pneumonia in late December 2019 in Wuhan, China.^1^ The outbreak spread quickly, and the WHO declared it as a public health emergency of international concern on January 30, 2020 and as a pandemic on March 11, 2020.^2^ As of May 29, 2020, the pandemic has infected 5,657,529 people and caused 356,254 deaths globally.^2^ As of this day, Bhutan had 33 confirmed cases of COVID-19, all of whom were imported. The first and the second cases were American tourist^3^: the first was airlifted and the second has recovered and left the country. The rest were Bhutanese studying or living abroad who recently returned from the United Kingdom (two), the United States (one), the Middle East (29), and India (one). Except the first case, all other cases were either asymptomatic or mild in clinical presentation, and none progressed to severe disease.

The most recognized mode of transmission of COVID-19 is by respiratory droplets and droplet contact, although several debates on questionable airborne transmission continues.^4–6^ During the early periods of the outbreak, the WHO confirmed human-to-human transmission of the virus and a preliminary reproducible number (*R*_0_) of 1.4–2.5 was estimated.^7^ The basic *R*_0_ in an analysis of early transmission dynamics in Wuhan was estimated to be 2.2 (95% CI: 1.4–3.9).^8^ When travel restriction was enforced, the median daily *R*_0_ in Wuhan declined from 2.35 (95% CI: 1.15–4.77) to 1.05 (0.41–2.39) after a week.^9^ Reports also indicated that COVID-19 has a higher effective *R*_0_ than SARS with a comparable fatality rate.^10^ A report on a familial cluster of COVID-19 cases in China seems to indicate easy transmissibility of the disease even by asymptomatic cases.^11^ Data also suggested that COVID-19 is efficiently transmitted in the community,^12^ and the *R*_0_ of COVID-19 was reported to be greater than that for infleunza.^8^ In Taiwan, an average secondary clinical attack rate of 0.9% (95% CI: 0.7–1.5) was reported, with a higher attack rate in those exposed within 5 days of symptom onset (2.4%) than those exposed later (zero cases in 605 close contacts). The attack rate was also higher in family contacts (13.6%) and non-household contacts (8.5%) than healthcare or other contacts.^13^

Bhutan’s national preparedness and response plan for COVID-19 is constantly reviewed based on emerging evidence. The most unique feature of Bhutan’s strategy includes a mandatory 21-day facility quarantine for all incoming individuals. During the quarantine period, individuals were tested by RT-PCR on days 3–5 and 13–14 and a rapid antibody test on day 22 (on completion of quarantine). In addition, an individual was tested on arrival at the point of entry (if symptomatic) and anytime during quarantine (if onset of symptoms reported). Secondary transmission of COVID-19 among the close contacts of these asymptomatic or mild cases has been minimal. We describe these limited secondary transmissions by the asymptomatic to mild Bhutanese patients and attempt to explain this from different perspectives.

## METHODS

This is a descriptive study related to the first 27 COVID-19–confirmed cases in Bhutan. Ethical approval was not required for descriptive and noninterventional studies related to the COVID-19 pandemic.

At the time of detecting these cases, viral RNA was extracted from 140 μL of nasopharyngeal swab collected in universal transport medium using a QIAamp viral RNA mini kit (QIAGEN, Hilden, Germany). SARS-CoV-2 viral genome was detected with the WHO-supplied MolBiol RT-PCR kit (TIBMolBio, Berlin, Germany) that targets *E* and RdRp gene of SARS-CoV-2. The kit claimed a sensitivity of 3.8 and 5.5 RNA copies/µL for *E* and RdRp genes, respectively. The *E* and RdRp genes were amplified under the following PCR conditions: 50°C for 30 minutes, 95°C for 2 minutes, followed by 45 cycles of 95°C for 15 seconds, and 55°C for 30 seconds in the ABI 7500 Fast Dx RT-PCR system (Thermofisher, Waltham, MA). Samples with Ct values of ≤ 40 were considered positive.

A line list of the first 33 laboratory-confirmed COVID-19 patients in the country was prepared. Similarly, all contacts of the patients (mostly in facility quarantine) were followed and reviewed for onset of signs and symptoms and positivity for COVID-19 tests. The follow-up continued until the completion of the 21-day mandatory quarantine or more in all cases.

Of the 33 cases detected until May 30, 2020, contacts of the first 27 cases were followed up and analyzed. Contacts were classified as primary (individuals coming in some form of contact with the confirmed cases such as conveyance in the same cars/flights, encounter in clinics, serving meals, or providing housekeeping services in hotels) or secondary (individuals coming in contact with the primary contacts). Among the primary contacts, further risk stratification was made for definite high-risk contacts such as driving in the same car, sitting in adjacent seats on flights, family members, close friends, and roommates in quarantine facilities. Each of these high-risk contacts was described individually in regard to the onset of signs and symptoms and testing for COVID-19. Results are presented as simple numerical values, percentages, and descriptions of individual case status as relevant.

## RESULTS

Bhutan had 33 laboratory-confirmed cases of COVID-19 as of May 29, 2020. Of these, 22 (67%) were females, indicating a definite female predominance of infected people. All cases were imported, and there was no community transmission at the time of this study. Except the first two cases who were American tourists (a 79-year-old man and a 59-year-old woman), the rest were all Bhutanese and of young age-group with a mean age of 26.3 (range 16–33) years. Of the 27 cases followed up and included in this study, 14 (52%) were asymptomatic, 12 (44%) were mild, and one (4%) was moderate (later progressed to severe disease) in clinical presentation. Among those symptomatic, fever, sore throat, loss of smell, and gastrointestinal presentation were the common symptoms. The first and the second cases had 73 and 97 primary contacts, respectively, and equal number of secondary contacts (224) because they traveled together. From the third case, a mandatory 21-day facility quarantine was instituted, and positive cases did not have any secondary contacts because all people traveling together in the same flight were considered primary contacts. In total, the 27 cases had 1,095 primary contacts and 448 secondary contacts. Of the primary contacts, there were 75 definite high-risk contacts among the primary contacts. The details of the confirmed cases are presented in Table 1.

**Table 1:** Details of the first 27 COVID-19–confirmed cases in Bhutan

Cases	Age (years)	Gender	Case origin	Disease category	Main symptoms	Primary contacts	Secondary contacts	High-risk contacts
C0001	76	M	Tourist (USA)	Moderate/severe	Gastrointestinal	73	224	5
C0002	59	F	Tourist (USA) (partner of C0001)	Asymptomatic	–	97	224	3
C0003	20	F	Bhutanese (London)	Mild	Fever and chills	31	0	2
C0004	19	F	Bhutanese (London)	Mild	Anosmia	28	0	1
C0005	16	F	Bhutanese (New York)	Asymptomatic	–	16	0	2
C0006	24	F	Bhutanese (Dubai)	Mild	Sore throat	24	0	3
C0007	24	M	Bhutanese (Doha)	Mild	Anosmia	14	0	2
C0008	27	M	Bhutanese (Dubai)	Asymptomatic	–	24	0	2
C0009	27	F	Bhutanese (Dubai)	Asymptomatic	–	24	0	2
C0010	25	M	Bhutanese (Kuwait)	Asymptomatic	–	37	0	3
C0011	27	F	Bhutanese (Dubai)	Mild	Fever and sore throat	39	0	3
C0012	33	M	Bhutanese (Dubai)	Asymptomatic	–	36	0	4
C0013	23	F	Bhutanese (Abu Dhabi)	Mild	Fever and headache	36	0	2
C0014	32	M	Bhutanese (Doha)	Mild	Fever, sore throat, and body ache	36	0	7
C0015	26	F	Bhutanese (Abu Dhabi)	Mild	Sore throat and diarrhea	19	0	2
C0016	31	F	Bhutanese (Dubai)	Asymptomatic	–	65	0	2
C0017	29	F	Bhutanese (Abu Dhabi)	Asymptomatic	–	65	0	3
C0018	24	F	Bhutanese (Dubai)	Asymptomatic	–	65	0	2
C0019	27	F	Bhutanese (Dubai)	Asymptomatic	–	65	0	3
C0020	30	M	Bhutanese (Doha)	Mild	Fever and diarrhea	37	0	3
C0021	29	F	Bhutanese (Doha)	Asymptomatic	–	36	0	5
C0022	30	F	Bhutanese (Abu Dhabi)	Asymptomatic	–	45	0	3
C0023	28	F	Bhutanese (Abu Dhabi)	Asymptomatic	–	45	0	2
C0024	34	F	Bhutanese (Dubai)	Asymptomatic	–	36	0	2
C0025	23	M	Bhutanese (Kuwait)	Mild	Nose block and anosmia	30	0	2
C0026	29	M	Bhutanese (Doha)	Mild	Fever and sore throat	36	0	3
C0027	29	M	Bhutanese (Doha)	Mild	Nasal irritation	36	0	2
Total						1,095	448	75

F = Female; M = male.

**Figure 1.**
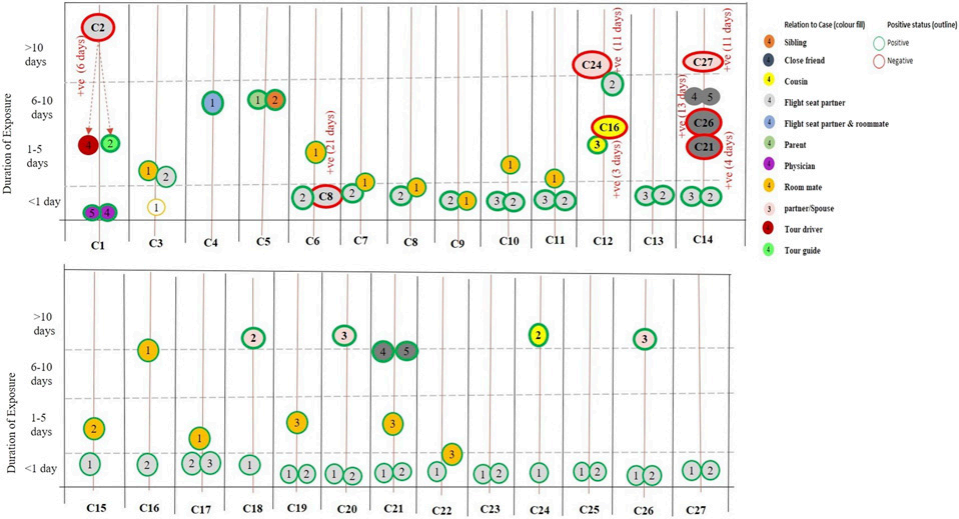
Relation between confirmed cases and contacts with time to positivity after last contact.

Every individual in description had been tested a minimum of three times with RT-PCR, unless they have turned positive before the next scheduled testing. From all these contacts, transmission occurred only in seven high-risk contacts. Therefore, the overall secondary transmission rate among the high-risk contacts was 9.0% (7/75), and that among the primary contacts was 0.6% (7/1,095), and none (0/448) among the secondary contacts. Of the seven positive cases, six of them tested positive with the normal incubation period (14 days) from last contact with a confirmed case and one tested positive on day 21 of exposure (Figure 1).

Definite close contacts with high risk for transmission of the virus from confirmed cases, their contact details, and transmission status are individually detailed in Table 2. Secondary transmission occurred commonly in partners/spouse (4/7), close friends (2/7), and flight seat partner (1/7). The observed Ct value of the sample and the presence of symptoms did not seem to affect the occurrence of secondary transmission.

**Table 2:** High-risk contacts and their SARS-CoV-2 transmission status

Case	High-risk contacts	Contact description	Level of contact	COVID-19 transmission status
C0001	5	Partner	Traveled together in flight, cruise, and car	Asymptomatic but positive after 6 days of last contact (C0002)
		Tour driver	Chauffeured the patient for 4 days	Asymptomatic and tested negative in between and on 28 days of last contact and discharged from quarantine
		Tour guide	Guided the patient for 4 days	
		Physician 1	Examined and talked with the patient > 2 hours. In close proximity, the patient had no face mask	
		Physician 2		
C0002	3	Tour driver	Common contacts of cases C0001 and C0002 because case C0002 was the partner of case C0001	
		Tour guide		
C0003	2	Roommate	Traveled in the same flight from London and spent three nights together in quarantine	
		Flight seat partner	Traveled in the same flight from Singapore	
C0004	1	Flight seat partner and roommate	Roommate in quarantine for 10 days	
C0005	2	Mother	Lived together in New York, traveled in the same flight to Bhutan, and spent two nights together in quarantine	
		Brother		
C0006	3	Roommate	Roommate in quarantine for three nights	
		Flight seat partner	Traveled together from Dubai to Bhutan	
		Flight seat partner		Asymptomatic but tested positive on day 21 testing (C008)
C0007	2	Roommate	Traveled together from Doha to Bhutan	Asymptomatic and tested negative in between and on 21 days of last contact and discharged from quarantine
		Flight seat partner	Traveled together from Doha to Bhutan	
C0008	2	Roommate	Roommate in quarantine for 21 days	
		Flight seat partner	Traveled together from Dubai to Bhutan	
C0009	2	Roommate	Roommate in quarantine for 21 days	
		Flight seat partner	Traveled together from Dubai to Bhutan	
C0010	3	Roommate	Roommate in quarantine for three nights	
		Flight seat partner	Traveled together from Kuwait	
		Flight seat partner		
C0011	3	Roommate	Roommate in quarantine for 1 day	
		Flight seat partner	Traveled together from Dubai to Bhutan	
		Flight seat partner		
C0012	4	Spouse	Traveled together and quarantined in the same facility for three nights	Asymptomatic and tested positive on day 11 of quarantine (C0024)
		Flight seat partner	Traveled together from Dubai to Bhutan	Asymptomatic and tested negative in between and on 21 days of last contact and discharged from quarantine
		Cousin	Stayed and traveled together from Dubai to Bhutan	Asymptomatic and tested positive on day 3 (C0016)
		Cousin		Asymptomatic and tested negative in between and on 21 days of last contact and discharged from quarantine
C0013	2	Flight seat partner	Traveled together from Dubai to Bhutan	
		Flight seat partner		
C0014	7	Spouse	Traveled together and quarantined in the same facility for two nights	Symptomatic and tested positive on day 11 of quarantine (C0027)
		Flight seat partner	Traveled together from Doha to Bhutan	Asymptomatic and tested negative in between and on 21 days of last contact and discharged from quarantine
		Flight seat partner	Traveled together from Doha to Bhutan	
		Close friend	Lived together in Doha and traveled together from Doha to Bhutan	
		Close friend		
		Close friend		Asymptomatic but tested positive on day 4 of quarantine (C0021)
		Close friend		Asymptomatic but tested positive on day 13 of quarantine (C0026)
C0015	2	Roommate	Roommate in quarantine for three nights	Asymptomatic and tested negative in between and on 21 days of last contact and discharged from quarantine
		Flight seat partner	Traveled together from Dubai to Bhutan	
C0016	2	Flight seat partner and roommate	Lived together, traveled on same flight and quarantined in same room	
		Flight seat partner	Traveled together from Dubai to Bhutan	
C0017	3	Flight seat partner	Traveled together from Dubai to Bhutan	
		Flight seat partner		
		Roommate	Roommate in quarantine for three nights	
C0018	2	Spouse	Traveled together and quarantined in the same room for two nights	
		Flight seat partner	Traveled together from Dubai to Bhutan	
C0019	3	Flight seat partner	Traveled together from Dubai to Bhutan	
		Flight seat partner		
		Roommate	Roommate in quarantine for three nights	
C0020	3	Spouse	Traveled together and quarantined in the same room for two nights	
		Flight seat partner	Traveled together from Doha to Bhutan	
		Flight seat partner		
C0021	5	Flight seat partner	Traveled together from Doha to Bhutan	
		Flight seat partner		
		Roommate	Roommate in quarantine for three nights	
		Close friend	Lived and traveled together from Doha to Bhutan	
		Close friend		
C0022	3	Flight seat partner	Traveled together from Dubai to Bhutan	
		Flight seat partner		
		Roommate	Roommate in quarantine for 11 days	
C0023	2	Flight seat partner	Traveled together from Dubai to Bhutan	
		Flight seat partner		
C0024	2	Flight seat partner	Traveled together from Dubai to Bhutan	
		Cousin	Lived and traveled together from Dubai to Bhutan	
C0025	2	Flight seat partner	Traveled together from Kuwait to Bhutan	
		Flight seat partner		
C0026	3	Spouse	Traveled together and quarantined in the same room for 12 days	
		Flight seat partner	Lived together and traveled together from Doha to Bhutan	
		Flight seat partner		
C0027	2	Flight seat partner	Traveled together from Doha to Bhutan	
		Flight seat partner		

## DISCUSSION

Rapidly increasing cases of COVID-19 worldwide with shortening durations between doubling numbers of confirmed cases in many countries seem to indicate high transmissibility of COVID-19. Presymptomatic transmissions with cluster transmissions also suggested easy transmissibility even through vocal activities such as singing and choir groups.^14,15^ By contrast, our study argues that asymptomatic or mild cases may not transmit the virus easily. The limited secondary transmission of SARS-CoV-2 presented in our study has been deduced from observing and testing the close contacts of laboratory-confirmed COVID-19 patients up to 21 days or more in strict facility quarantine. All contacts have undergone constant monitoring for the onset of symptoms and scheduled testing (at least three RT-PCR tests) including the antibody testing at the end of the 21-day facility quarantine. Therefore, for an accepted mean incubation period of 5.2 (range 2–14) days^8^ for COVID-19 disease, a quarantine period of 21 days or more followed by testing would have not missed any cases, and the findings of this study hold much value.

This observation may be attributed to many factors such as clinical severity, race, younger patients, and living in high altitude which were opined to be protective against transmission and severity of COVID-19 clinical manifestations. In addition, the preventive measures such as using face masks, cough etiquette, and hand hygiene, which were already being widely promoted, could have had positive impact on preventing the transmission of the virus. Clinically, patients who are asymptomatic or mild with none or minimal cough, sneezing, or respiratory distress (with no labored breathing) probably do not transmit the virus easily because of the limited respiratory secretions or droplets expelled into the air. Racial differences in COVID-19 susceptibility and disease severity have been described in the Americas with African American individuals and, to a lesser extent, Latino individuals bearing a disproportionate burden of COVID-19–related outcomes.^16^ Such racial and ethnic effects may be relevant to the Bhutanese ethnicity who are uniquely adapted to the Himalayas. Epidemiological data from Tibet and high-altitude regions of Bolivia and Ecuador compared with lowland suggested that high-altitude inhabitants (+2,500 m above sea level) are less susceptible to develop severe adverse effect in acute SARS-CoV-2 virus infection. This was likely because of physiological adaptations counterbalancing the hypoxic environment of high altitude that protect from severe impact of acute SARS-CoV-2 virus infection.^17^ Bhutan has human settlement at altitudes of up to 3,700 m above mean sea level,^18^ and this relation to high altitude may provide an explanation for almost all asymptomatic to mild cases among all the 33 cases. In another Tibetan study, 36 of the 67 (54%) COVID-19 patients were asymptomatic, with only seven (10%) progressed to severe disease and recovered with no death. In addition, imported cases of COVID-19 in Tibetan patients were reported to be generally mild with absence of fever or radiologic abnormalities.^19^ This observation is also in concurrence with imported cases of COVID-19 in Bhutan, with all the 33 Bhutanese cases being asymptomatic to mild. Plausible explanations for asymptomatic to mild cases in the Bhutanese patients are age (all young patients, the oldest being 33 years) and universal childhood vaccinations with Bacillus Calmette-Guerin (BCG) and oral polio vaccine (OPV) vaccinations as part of the Expanded Program on Immunization, with high vaccine coverage. This conclusion is in line with the finding that countries with BCG and OPV vaccination had lesser cases and low mortality from COVID-19.^20^ Analysis on BCG concluded that countries without universal policies of BCG vaccination (Italy, the Netherlands, the United States) had been severely affected compared with countries with universal and long-standing BCG policies. BCG vaccination was also found to be associated with the number of reported COVID-19 cases in a country.^21,22^

A modeling in Singapore has shown that implementing a combined intervention of quarantining infected individuals and their family members, workplace distancing, and school closure after community transmission ensues could substantially reduce the number of SARS-CoV-2 infections.^23^ Bhutan’s institution of these measures even before the onset of community transmissions has been highly effective in preventing the transmission and spread into the community. Bhutan received its first case of COVID-19 only on March 5, 2020, after a lot of planning and advocacy. By then, people have been educated on preventive measures. Therefore, to a certain extent, it is likely that the contacts of the cases would have been practicing all the preventive measures, which could mitigate the risk of transmission during their contact. In Tianjin (China), cluster outbreaks in families, workplace, transport vehicles, and other public places were reported. These findings emphasized that special attention should be paid to the cases from the same family, same workplace, or other places where clustering is likely to occur, and the epidemiological investigation should be carried out timely to confirm the cluster. It also recommended that the close contacts of the patients should be transferred to an assigned observation place in time for single-room isolation.^24^ These actions have been the key features of the COVID-19 prevention and control response in Bhutan and have been proven to be effective, with minimal community transmission to date.

This study is not short of limitations, the main related to the inclusion of primary and secondary contacts. All the cases in description were imported, and all secondary contacts were related to the first two cases. Beginning with the third case, all contacts were invariably included as primary contacts and put under mandatory facility quarantine on arrival at the point of entry into the country. This inclusion potentially biased the cohort and may have impacted the transmission rate. In addition, viral load could not be performed, and transmission dynamics based on Ct values and presence or absence of symptoms may need to be interpreted with caution.
